# Facile Fabrication of Self‐Assembly Functionalized Polythiophene Hole Transporting Layer for High Performance Perovskite Solar Cells

**DOI:** 10.1002/advs.202002718

**Published:** 2021-01-06

**Authors:** Chi‐Yuan Chang, Hsin‐Hsiang Huang, Hsinhan Tsai, Shu‐Ling Lin, Pang‐Hsiao Liu, Wei Chen, Fang‐Chi Hsu, Wanyi Nie, Yang‐Fang Chen, Leeyih Wang

**Affiliations:** ^1^ Center for Condensed Matter Sciences National Taiwan University No. 1, Sec. 4, Roosevelt Rd. Taipei 10617 Taiwan; ^2^ Department of Physics National Taiwan University No. 1, Sec. 4, Roosevelt Rd. Taipei 10617 Taiwan; ^3^ Materials Science Division and Center for Molecular Engineering Argonne National Laboratory Lemont IL 60439 USA; ^4^ Department of Material Science and Engineering National Taiwan University No. 1, Sec. 4, Roosevelt Rd. Taipei 10617 Taiwan; ^5^ Center for Integrated Nanotechnologies, Materials Physics and Application Division Los Alamos National Laboratory Los Alamos NM 87545 USA; ^6^ Pritzker School of Molecular Engineering University of Chicago Chicago IL 60637 USA; ^7^ Department of Material Science and Engineering National United University 1, Lienda Miaoli 36003 Taiwan; ^8^ Institute of Polymer Science and Engineering National Taiwan University No. 1, Sec. 4, Roosevelt Rd. Taipei 10617 Taiwan

**Keywords:** hole extraction layer, out‐of‐plane orientation, self‐assembled

## Abstract

Crystallinity and crystal orientation have a predominant impact on a materials’ semiconducting properties, thus it is essential to manipulate the microstructure arrangements for desired semiconducting device performance. Here, ultra‐uniform hole‐transporting material (HTM) by self‐assembling COOH‐functionalized P3HT (P3HT‐COOH) is fabricated, on which near single crystal quality perovskite thin film can be grown. In particular, the self‐assembly approach facilitates the P3HT‐COOH molecules to form an ordered and homogeneous monolayer on top of the indium tin oxide (ITO) electrode facilitate the perovskite crystalline film growth with high quality and preferred orientations. After detailed spectroscopy and device characterizations, it is found that the carboxylic acid anchoring groups can down‐shift the work function and passivate the ITO surface, retarding the interface carrier recombination. As a result, the device made with the self‐assembled HTM show high open‐circuit voltage over 1.10 V and extend the lifetime over 4,300 h when storing at 30% relative humidity. Moreover, the cell works efficiently under much reduced light power, making it useful as power source under dim‐light conditions. The demonstration suggests a new facile way of fabricating monolayer HTM for high efficiency perovskite devices, as well as the interconnecting layer needed for tandem cell.

## Graphical abstract

Self‐assembled P3HT‐COOH is an excellent hole extraction layer to fabricate robust, high‐performance, and extremely reproducible perovskite solar cells. The well‐aligned self‐assembled P3HT‐COOH generates a dipole layer between indium tin oxide and perovskite, substantially retarding interface charge recombination and producing highly sensitive devices to dim light. The enhanced crystallinity and preferred out‐of‐plane orientation play a key role to suppress the device degradation process.

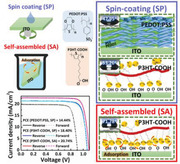

Organometal halide perovskite materials have aroused tremendous interest in recent years because of their excellent semiconductor properties such as high absorption coefficient, bandgap tunability, defect tolerance, long carrier lifetime, and diffusion length.^[^ ^1^, ^2^, ^3^, ^4^ ^]^ These characteristics make perovskites one of the most promising candidate for next‐generation thin film photovoltaics materials. The power conversion efficiency (PCE) of perovskite photovoltaics has rapidly surged from 3.8% to 25.2% in the past decade.^[^ ^5^, ^6^, ^7^, ^8^ ^]^ Among those popular perovskite device architectures, the p–i–n device employing polymer contact layers has gain substantial interests because all the layers can be fabricated at low temperature via solution method, and it allows for flexible device fabrications. In 2013, Snaith et al. adopted poly(3,4‐ethylenedioxythiophene) polystyrene sulfonate (PEDOT:PSS) as the hole transporting material (HTM) to build flexible planar perovskite solar cells (PSCs) on polymer substrates with a PCE of around 6%, making perovskite photovoltaic attractive technology for flexible opto‐electronics.^[^ ^9^ ^]^ However, the PEDOT:PSS is strongly acidic and hydrophilic that can corrode the indium tin oxide (ITO) electrode and accelerate the moisture induced degradation, resulting in rapid dropping of the devices’ PCE.^[^ ^10^ ^]^ It was also reported that the diffusion of indium and tin ions into the perovskite layer without barrier at the interface can significantly degrade the device.^[^ ^11^ ^]^ More recently, poly[bis(4‐phenyl)(2,4,6‐trimethylphenyl)‐amine] (PTAA) has been widely used as HTM that can extract holes efficiently,^[^ ^12^, ^13^ ^]^ yet these polymers are still costly comparing to PEDOT:PSS. In addition, other HTMs have been prepared by depositing triphenylamine‐based molecules on a transparent conductive oxide.^[^ ^14^, ^15^, ^16^, ^17^ ^]^ However, those organic HTMs usually possess lower carrier mobility (10^−2^ –10^−4^ cm^2^ V^−1^ s^−1^) and required dopants, such as lithium bis(trifluoromethanesulfonyl)imide (Li‐TFSI) and 4‐tert‐butylpyridine (TBP), to improve conductivity.^[^ ^12^, ^18^, ^19^ ^]^ However, the dopants are also hydrophilic compounds that can often expedite the perovskite layer degradations when being exposed to a humid environment.^[^ ^20^ ^]^ To improve stability, several inorganic HTMs such as nickel oxide (NiO*_x_*)^[^ ^21^, ^22^, ^23^ ^]^ and copper oxide (CuO*_x_*)^[^ ^24^, ^25^ ^]^ were developed. However, the inorganic HTMs have several drawbacks such as severe interface recombination and low compatibility with flexible devices. To address the above‐mentioned issues and fully realize the advantages of PSCs, an alternative strategy to prepare effective HTM is urgently needed.

There have been a few encouraging reports on using conjugated polyelectrolyte as HTM recently that improved the charge transport properties in the perovskite devices. For instance, Zhang et al. introduced thiophene‐based polyelectrolyte (P3CT‐Na) as HTM in perovskite solar cells achieving PCE over 16%, which was 20% improvement compared with PEDOT:PSS devices.^[^ ^26^ ^]^ Besides, by controlling the aggregation in polyelectrolytes based HTM film where much uniform perovskite layer can grow, Li et al. showed an enhanced performance of PSCs fabricated on both rigid and flexible substrates.^[^ ^27^ ^]^ Zhang et al. demonstrated that using dopant‐free polyelectrolyte (P3CT‐BN) as HTM can reduce interfacial recombination to improve the cell stability.^[^ ^28^ ^]^ More recently, Li et al. showed that using P3CT‐Rb as HTM led to a large open circuit voltage (*V*_OC_) of 1.144 V.^[^ ^29^ ^]^ In addition, the P3HT with carboxylic groups in the ends of the main chain was synthesized by Lohwasser et al. and demonstrated its potential anchor onto mesoporous TiO_2_ as a polymer sensitizer in a solid‐state dye‐sensitized solar cell.^[^ ^30^ ^]^ Arbati et al. demonstrated a combination of reduced graphene oxide (rGO) nanosheets with carboxyl groups grafted with P3HT to modify the morphology of active layers in the organic photovoltaic devices.^[^ ^31^ ^]^ These reports demonstrate the potential of polyelectrolyte as promising HTMs for efficient charge collections in high performance photovoltaics with improved device operational stability. However, several fundamental questions remain open, such as the role of crystallinity, molecular structure, and their interface properties when growing MAPbI_3_ on the polymer thin films, these are all critical issues that impact the performance and stability.

Here, we demonstrate a new self‐assembled monolayer, poly[3‐(6‐carboxyhexyl)thiophene‐2,5‐diyl] (P3HT‐COOH), on the conducting oxide electrode as HTM to interface with the photoactive perovskite layer, which facilitate the perovskite layer growth and suppress the interface recombination. Especially, perovskite film grown on the self‐assembled P3HT‐COOH layer exhibits greatly enhanced crystallinity with characteristic X‐ray diffraction peak splitting similar to that from the single crystal. Utilizing the synchrotron grazing incident wide‐angle X‐ray scattering (GIWAXS) technique, we find the perovskites layer shows slight preference for being oriented with (002) planes parallel to the substrate. This results in high performance, reproducible, and hysteresis free PSC with high efficiency over 20%. Notably, the *V*_OC_ substantially increase from 0.86 ± 0.06 to 1.09 ± 0.01 V. Extensive morphology, optical spectroscopy, and device characterizations reveal a suppressed interfacial recombination in the perovskite device prepared on the P3HT‐COOH HTM. Because of the greatly suppressed dark current, we demonstrate a sensitive photo detector under low light conditions. The enhanced crystallinity and preferred out‐of‐plane orientation play a key role to suppress the device degradation process, thus extend the device lifetime over 4300 h when storing at 30% relative humidity.

First, we fabricate self‐assemble (SA) P3HT‐COOH monolayer on ITO as HTM. To directly compare the conjugated polyelectrolyte P3HT‐COOH with the benchmark material PEDOT:PSS as HTM and their chemical structures, here we also deposited the HTMs on ITO substrates with commonly used spin‐coating (SP) approach (**Figure** **1**a). We first measure the contact angles of water on these three HTMs with SP method on ITO substrates to determine the surface hydrophobicity. Figure 1b is the PEDOT:PSS film (top panel) and P3HT‐COOH film (bottom panel) fabricated by SP method, the contact angles are 47.95° and 53.20^o^, respectively, which suggest both HTMs have similar surface energy. This is because both surfaces have partial carboxylic or sulfonate group that are randomly oriented on the surface, leading to more hydrophilic surface.^[^ ^32^, ^33^ ^]^ On contrary, the contact angle for SA P3HT‐COOH film increases to 71.78^o^. This is because the carboxylic group in P3HT‐COOH polymer chains can freely rotate and adjust their carboxylic group to ITO surface in the liquid phase that consequently increases the anchoring density of carboxylic groups on the ITO surface and forming a highly oriented electric dipole layer, leaving less density of hydrophilic groups pointing toward the surface of P3HT‐COOH film (Figure 1c). We use photoluminescence (PL) to probe the quality of the interface. As shown in Figure S1 (Supporting Information), the P3HT‐COOH (SA) layer yields a much higher PL intensity compared to the spin coated P3HT‐COOH layer, indicating the SA P3HT‐COOH film possesses a lower defect density.^[^ ^27^, ^34^ ^]^ Note that, the arrangement of carboxylic groups on ITO has been reported to induce an interfacial dipole and passivate the ITO surface which can facilitate the holes collection and blocking the carrier recombination.^[^ ^35^ ^]^ It may also arise from weak P3HT aggregation and the well oriented polymer chain arrangement with a coil‐like conformation.^[^ ^27^ ^]^

**Figure 1:**
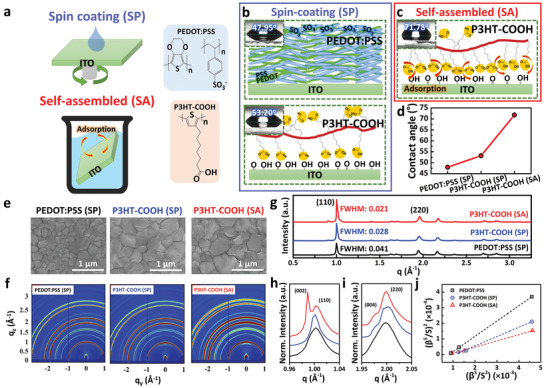
a) Spin coating and self‐assembly route to prepare PEDOT:PSS and P3HT‐COOH on ITO substrates. Schematic illustration for spin coated polymer thin film of b) PEDOT:PSS (top), P3HT‐COOH (bottom), and c) self‐assembled P3HT‐COOH. The insets are the contact angle images of deionized water on fabricated HTMs from (b)–(c). d) Contact angle plot with HTMs with different fabrication methods. e) Surface SEM images and f) GIWAXS patterns showing the evolution of the three perovskite grain types under different HTMs. g) GIWAXS line‐cut from f with their FWHM on the characteristic peaks of (110) plane in MAPbI_3_ on three different HTMs. Zoom‐in profiles for h) (110) and i) (200) peaks. j) Integral breadth (IB) analysis using Halder–Wagner plots, where *β* is the integral breadth of the diffraction peak and *S* is defined as $S\;=\frac{2sin\theta }{\lambda }\;$.

After understanding the surface properties of these HTMs layers, we then fabricate the perovskite photoactive layer on the HTMs and characterize their morphology and crystallinity (Figure 1e–j). From the scanning electron microscope (SEM) images of the perovskite grown on different HTMs in Figure 1e, we find the crystal grain of the perovskite layer can grow larger when the surface energy of the HTMs increases. The average perovskite grain size on these HTMs (PEDOT:PSS (SP), P3HT‐COOH (SP), and P3HT‐COOH (SA)) are 155, 394, and 409 nm, respectively (Figure S2, Supporting Information). This might due to the non‐wetting surface of P3HT‐COOH layer not only can assist the crystal formation but also can form high‐quality perovskites on top of it. The observation is consistent with the literature report^[^ ^14^ ^]^ where a nonwetting surface can assist the crystal formation. In general, a large crystal grain is beneficial to the creation of long‐lived carriers to enable efficient charge transport (Figure S3, Supporting Information).^[^ ^36^ ^]^

We further characterized the crystallinity and crystal orientation of these perovskite film with GIWAXS, as illustrated in Figure 1f and their GIWAXS line‐cut in Figure 1g along out‐of‐plan direction. After indexing the peaks, we found all the samples follow the tetragonal phase signatures as expected in MAPbI_3_ samples,^[^ ^37^ ^]^ without the non‐converted precursor or other impurity phase. The full‐width‐at‐half‐maximum (FWHM) of the (110) peak at *q* ≈ 1.0 Å^−1^ are 0.041, 0.028, and 0.021 Å^−1^, corresponding to crystallite sizes of 14, 20, and 27 nm for the perovskite films grown on the PEDOT:PSS (SP), P3HT‐COOH (SP), and P3HT‐COOH (SA) surface, respectively. Notably, the grain size observed by SEM is much larger than the crystallite sizes extracted by GIWAXS, indicating that the perovskite grains observed from SEM may be formed by small crystallites.^[^ ^38^ ^]^ Moreover, we performed detail analysis on GIWAXS patterns and peak indexing for the perovskite thin film grown on these HTMs (Figure 1h–i), the P3HT‐COOH (SA) thin film exhibits the splitting in (002)/(110) and (004)/(220) planes which are observed in single crystals of MAPbI_3_.^[^ ^39^ ^]^ In sharp contrast, the perovskite thin films fabricated on PEDOT:PSS (SP) and P3HT‐COOH (SP) only exhibit broadened (110) and (220) peaks that covers the peak splitting signatures. To evaluate the crystallite size and strain distribution, we performed a detailed structural analysis using an integral breadths (IB) analysis of all the diffraction planes based on the Halder–Wagner equation (Figure 1j). The coherence length, which describes the average crystallite size, was obtained from the slope of the linear fit of the Halder–Wagner plot. The observed reduction in the slope that resulted from an increase in the average crystallite size. These results suggest that the perovskites thin film fabricated on P3HT‐COOH (SA) exhibits enhanced crystallinity compare with sample grown on PEDOT:PSS (SP) and P3HT‐COOH (SP).

To further investigate the crystalline orientation of the perovskite layer grown on different HTMs, we plot azimuthal profiles of the X‐ray scattering intensity for the feature at *q* ≈ 1.0 Å^−1^ in Figure S4 in the Supporting Information. We find that the highest scattering intensity is close to *χ* = 0^o^, and the reflection rings of perovskite film grown on P3HT‐COOH (SA) surface have a stronger nonuniform intensity distribution in the azimuthal direction. The resulting scattering patterns reveal two major characteristics for the perovskite films fabricated on different HTMs. The perovskite layer grown on P3HT‐COOH (SA) is more crystalline where the (002) plane are oriented most along out‐of‐plane direction, indicating the crystal domains are parallel to the substrate surface. In contrast, the perovskite grown on PEDOT:PSS films exhibit more isotropic rings, which indicates considerable randomness in the 3D orientation of the crystal domains. Note that previous works have suggested that the perovskite films with large grain are mostly oriented out‐of‐plane direction in parallel to the substrate surface, and such orientation can strongly impact the device performance.^[^ ^40^, ^41^ ^]^ Combining the high degree of crystallinity and preferable orientations in MAPbI_3_ grown on our SA layers, we expect a high performance from the perovskite solar cell fabricated by our samples.

**Figure** **2**a illustrates the device architecture with energy level for each component used in this study. It has been recognized that the highest occupied molecular orbit (HOMO) energy level of the polymer HTM is one of the key factors determining the *V*_OC_ of PSCs and a low‐lying HOMO is beneficial for a high‐*V*_OC_ in the solar cell.^[^ ^42^ ^]^ As shown in Figure 2a and Table S1 in the Supporting Information, the ionization potentials (*I*_p_) measured by photoelectron spectrometer (Figure S5, Supporting Information), the carboxyl groups at the alkyl chain‐ends in the P3HT based HTMs have negligible influence on the energy levels of the conjugated backbones, where the *I*_p_ of a P3HT‐COOH bulk (−5.10 eV) is comparable to that of P3HT bulk (−5.11 eV). Interestingly, both SP and SA layers of P3HT‐COOH exhibit a noticeably deepened *I*_p_ of −5.32 and −5.42 eV, respectively. We attribute the deeper HOMO in thin P3HT‐COOH films to the reduced polymer aggregations (see UV–vis absorption analysis in Figure S6 in the Supporting Information).^[^ ^43^ ^]^ We then deposited the photoactive MAPbI_3_ films on these substrates and assemble the PSCs. Here, the MAPbI_3_ layers have a comparable thickness (Figure S7, Supporting Information) and exhibit almost overlapped absorption spectra (Figure S8, Supporting Information), so that the light‐harvesting capability of the perovskite layer is not the main factor causing the difference in these three PSCs. Note the thickness of P3HT‐COOH (SP) layer was kept around 1 nm, comparable to that of the SA monolayer. Figure 2b and Figure S9 (Supporting Information) display the current density–voltage (*J–V*) characteristics and steady‐state output of the PSCs using multiple approach for fabrication of HTM, respectively. The photovoltaics device based on self‐assembled PEDOT:PSS exhibited an extremely poor performance due to an uneven surface and discontinuous coverage which would not be further discussed (see Supporting Information and Figures S10–S12 in the Supporting Information). For the spin‐coated PEDOT:PSS and P3HT‐COOH devices, the *V*_OC_ (1.06 V) and *J*_SC_ (21.46 mA cm^−2^) in P3HT‐COOH are higher than those in PEDOT:PSS devices (*V*_OC_ = 0.91 V and *J*_SC_ = 19.70 mA cm^−2^). This is benefitted from the deeper HOMO level in P3HT HTMs. Moreover, the self‐assembled P3HT‐COOH device further increased the *V*_OC_ to 1.10 V and *J*_SC_ to 22.83 mA cm^−2^. The solar cell statistics are summarized in Figure 2c and Table S2 (Supporting Information) by averaging over 60 devices. The P3HT‐COOH (SA) not only has the highest photovoltaic performance but also narrowest performance distribution. The external quantum efficiency (EQE) for these devices are shown in Figure 2d. The P3HT‐COOH devices have higher EQE than PEDOT:PSS device over the entire wavelength range. Interestingly, we observed the dip in EQE in range of 700 to 900 nm. This is due to the thickness of absorber layer which can affect the optical interference within the device stacking.^[^ ^44^ ^]^ Further study and improvement are indeed needed with our current study for increase absorber thickness. As a result, the integrated photocurrents from the EQE spectra for these PEDOT:PSS (SP), P3HT‐COOH (SP), and P3HT‐COOH (SA) are 18.23, 20.21, and 22.13 mA cm^−2^ which are in accordance with the experimental current density obtained from the *J–V* measurements.

**Figure 2:**
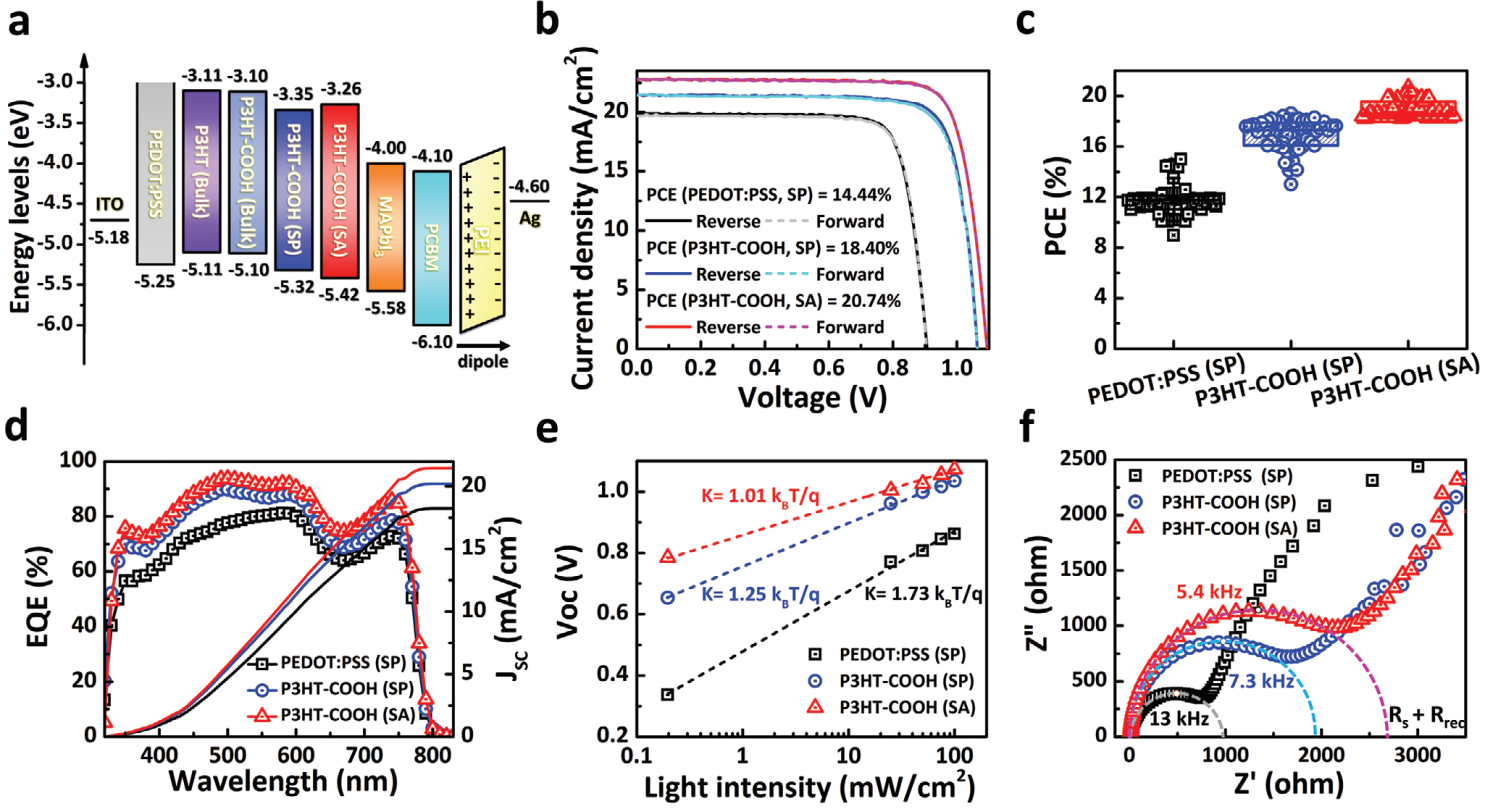
a) Energy level diagrams of MAPbI_3_ and various HTMs. b) *J–V* curves of PSCs using PEDOT:PSS or P3HT‐COOH as HTM prepared by either SP or SA method. c) Statistics of 60 devices fabricated with different HTMs as collected over 5 different batches. We note that all device parameters and the standard deviation, a metric for the reproducibility, improved. d) EQE Spectra of PSCs with different HTMs. e) *V*_OC_ versus light intensity and f) Nyquist plots of the PSCs fabricated with different HTMs.

In order to understand the carrier recombination processes during device operation, we tested the device response as a function of light intensities in the range of 0.2–100 mW cm^−2^. The relationship between *V*_OC_ and light intensity is also plotted (Figure 2e). When linear fitting the *V*_OC_ as a function of light power in log scale, we obtained a slope *K* = 1.01 *k*_B_ *T/q* (where *k*_B_ is the Boltzmann constant, *T* is absolute temperature, and *q* is elementary charge) for a P3HT‐COOH (SA) device, which suggests that a bimolecular recombination process dominates during device operation.^[^ ^45^, ^46^, ^47^ ^]^ Comparing the spin coated P3HT‐COOH (*K* = 1.25 *k*_B_ *T/q*) and PEDOT:PSS (*K* = 1.73 *k_B_T/q*) device, the higher *K* values in the latter cases are indicative of trap‐assisted recombination.^[^ ^45^, ^46^, ^47^ ^]^ To probe the recombination of photogenerated carriers during device operation, we conducted electrical impedance spectroscopy (EIS) measurements on these devices to investigate the charge transport and recombination across the HTM/MAPbI_3_ interface. All three Nyquist plots in Figure 2f obtained from PEDOT:PSS (SP), P3HT‐COOH (SP), and P3HT‐COOH (SA) devices exhibit two distinct charge transport regimes: (a) A high frequency semi‐circle originating from interface charge transport and recombination kinetics; (b) a low frequency semi‐circle arising predominately from slow ion migration.^[^ ^48^, ^49^ ^]^ Obviously, the PEDOT:PSS (SP) device has the smallest high frequency arc, corresponding to a small recombination resistance (*R*_rec_). This indicates a high charge recombination occurring at the PEDOT:PSS (SP)/MAPbI_3_ interface. On the other hand, both the P3HT‐COOH‐based PSCs have higher *R*_rec_ than PEDOT:PSS device and P3HT‐COOH (SA) cell has the largest the *R*_rec_, suggesting the monolayer of P3HT‐COOH at the interface can effectively diminish the recombination lose. Moreover, the lifetime of free carriers recombination can be estimated from the characteristic frequency at the highest point of the corresponding semicircle.^[^ ^50^ ^]^ They increase in the order of PEDOT:PSS (SP) (12 µs) < P3HT‐COOH (SP) (21 µs) < P3HT‐COOH (SA) (27.8 µs). The increased carrier lifetime is consistent with the reduced charge recombination. From these results, we believe the greatly suppressed recombination and efficient charge collection originate from the high‐quality perovskites layer deposited on ordered P3HT‐COOH (SA), which builds a clean interface.

The rise of Internet of Things (IoT) demands an efficient off‐grid energy provider for potentially billions of discrete sensing nodes.^[^ ^51^, ^52^ ^]^ Among all energy types including thermal, vibration, and photon energy, photon energy exhibits much higher energy density in the general environment and hence, photovoltaics devices which are suitable for environmental dim‐light energy harvesting become one of the most promising power providers for numerous IoT sensing nodes.^[^ ^53^ ^]^ However, the grand challenge of low‐light application is the high dark current noise, which would affect the photocurrent of PSCs under low‐light illumination, resulting in poor performance. While measuring the dark current, we realized that the P3HT‐COOH based PSCs maintains the photo‐response at much lower light intensity than the PEDOT:PSS device. The SA P3HT‐COOH HTM device shows ultralow dark current of 7.3 × 10^−8^ mA cm^−2^, which is three orders of magnitude lower than the PEDOT:PSS device (4.9 × 10^−5^ mA cm^−2^) at the reverse bias, because of the efficient dark current blocking and suppressed interface recombination (Figure S14, Supporting Information). It is thus an excellent candidate for ultra‐sensitive low light detection,^[^ ^54^ ^]^ as well as novel power sources for applications like indoor PSCs^[^ ^55^, ^56^ ^]^ and image sensor.^[^ ^57^ ^]^ Therefore, we collected the *J–V* curves of PSCs at various low light intensities. **Figure** **3**a–c shows the *J*–*V* characteristic curve s under light intensities from 0.9 nW cm^−2^ to 196 µW cm^−2^. The data reveal that the increase of illuminated light intensity from 0.9, 800 to 1490 nW cm^−2^ significantly shifted the *V_OC_* from 59, 487 to 653 mV for the SA‐cell, and from 9, 165 to 448 mV for the SP‐cell but hardly moved the *V*_OC_ of the PEDOT:PSS cell from 0 mV. Owing to the reciprocity relationship between *V*_OC_ and reverse bias saturated dark current^[^ ^58^ ^]^ described by following Equation: *V*_OC_ = *nkT/q·ln(I*_sc_ */I*_0_) (where *n* is the ideality Factor, *k* is the Boltzmann constant, *T* is the temperature in Kelvin, *q* is the elementary charge, *I*_sc_ is the photocurrent and, *I*_0_ is the reverse bias saturated dark current) and the suppressed dark current does contribute to the increase in *V*_OC_. This observation further evidences that the interfacial charge recombination which is significantly suppressed in the P3HT‐COOH based cells and, therefore, the photoinduced charge carriers can build up the photovoltage rapidly. We further plot *V*_OC_ and *J*_SC_ against logarithm of light intensity in Figure 3d,e to investigate the carrier recombination losses near open circuit. The P3HT‐COOH (SA) device maintain the slope of *K* = 1.08 *k*_B_ *T/q* in the linear *V*_OC_–Ln(power) curve whereas the plot slope decreased to *K* = 2.01 *k*_B_ *T/q* for P3HT‐COOH (SP). In contrast, the *V*_OC_ for the PEDOT:PSS (SP) device follows two linear regimes, one at high power with a slope of about *K* = 2.10 *k_B_T/q* and the other shows a much lower threshold for the change in slope of *V*_OC_ then deviate from linear relationship, which probably due to the high dark current. This observation further validates that the interfacial charge recombination is significantly suppressed in the P3HT‐COOH based cells. To compare with PEDOT:PSS (SP) and P3HT‐COOH (SP) cells, the photovoltage of the P3HT‐COOH (SA) devices are extremely sensitive to low light intensity (nW–µW range), indicating its potential application as a highly responsive low‐light detection.

**Figure 3:**
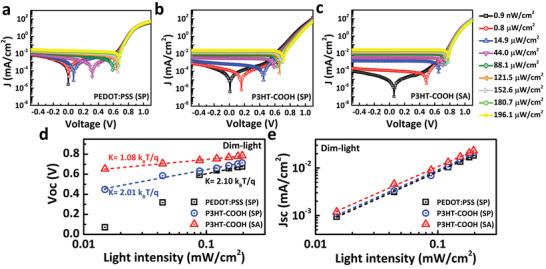
*J–V* curves of a) PEDOT:PSS (SP), b) P3HT‐COOH (SP), and c) P3HT‐COOH (SA) under dim light. d) The *V*_oc_ versus dim light intensity of the perovskite grown on different HTMs. The *J–V* curves of PSCs were measured at various low light levels using a LED lamp with a spectrum displayed in Figure S13 in the Supporting Information.

It is known that perovskite materials decomposition with moisture is one of the key factors that limits the device operational lifetime. Moreover, it has been reported that the diffusion of metal ions in ITO, such as indium and tin, into the perovskite layer can significantly degrade the PCE.^[^ ^11^ ^]^ To further examine device operation stability, we performed a series of un‐encapsulated solar cells stability test under relative humidity (RH), constant thermal and 1‐sun light intensity conditions. The specific parameter employed to evaluate the stability is the time when the device performance drops to 80% of the initial value (*T*_80_ lifetime). **Figure** **4**a,b is the device operation under different humidity conditions (RH 30% and 55%). In the RH of 30%, the P3HT‐COOH (SA) and P3HT‐COOH (SP) devices exhibit impressive *T*_80_ lifetime with *T*_80_ = 4300 and 2000 h, respectively. In sharp contrast, the PEDOT:PSS (SP) device only have *T*_80_ lifetime of less than 240 h. Moreover, we increase the environmental humidity to RH = 55%, the *T*_80_ lifetime for these three devices are all decrease dramatically.

**Figure 4:**
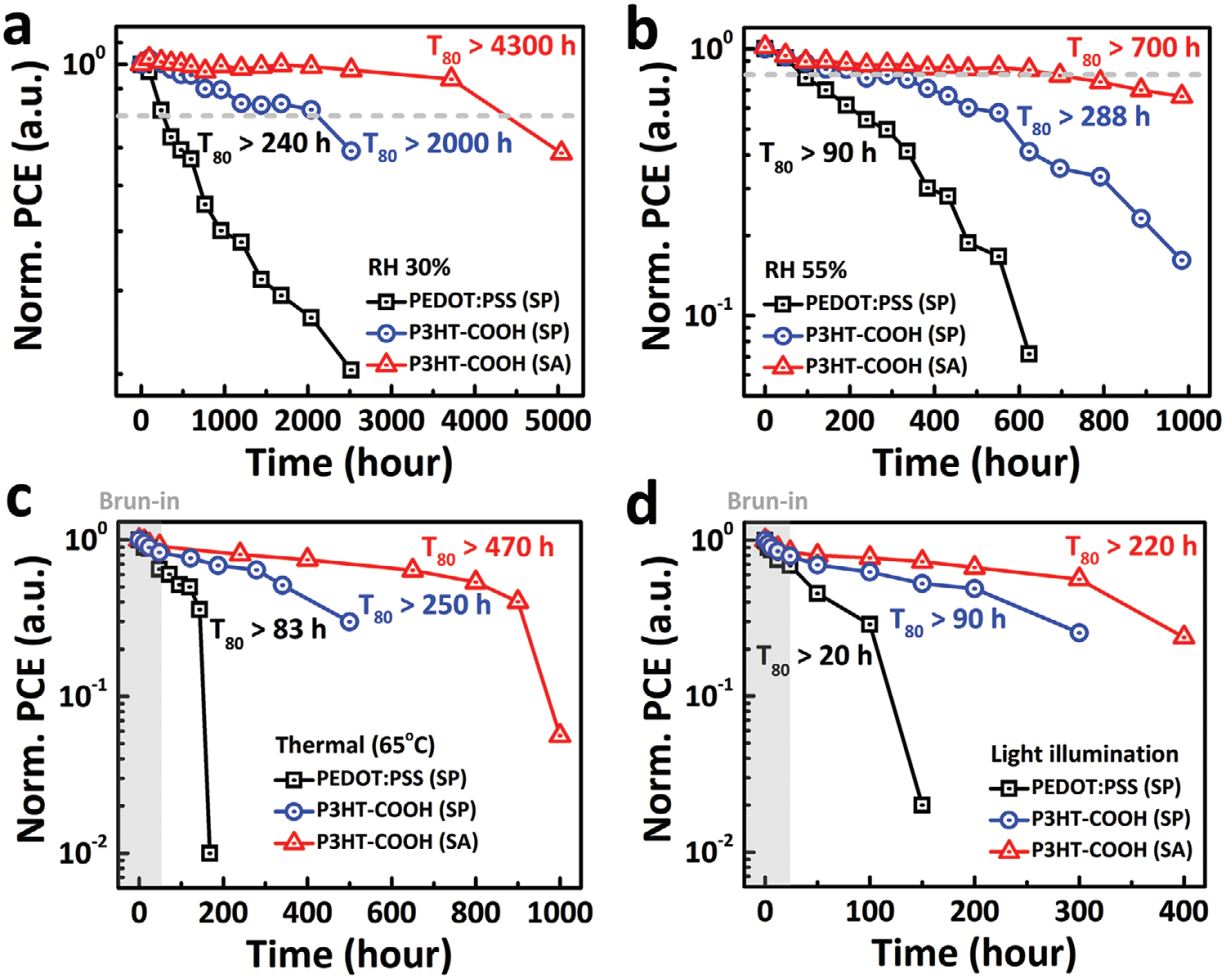
Stability test of PSCs with various HTMs stored under a) 30% and b) 55% relative humidity. c) Normalized PCE of devices under thermal testing at 65 °C in glove box and d) photostability test under constant 1‐sun illumination with a full spectrum AM1.5G source. All data were collected over ten devices for statistics (Figure S15, Supporting Information).

The P3HT‐COOH (SA) and P3HT‐COOH (SP) cells remain *T*_80_ lifetime of 700 and 288 h whereas the PEDOT:PSS (SP) device undergoes rapid degradation with *T*_80_ of 90 h for and losses its function within 600 h. This is attribute to the highly hydrophilic nature and strongly acidic ‐SO_3_H groups in PEDOT:PSS can induce a fast water uptake and further cause the material decompositions which results in rapid dropping of PCE.^[^ ^10^, ^59^, ^60^ ^]^ We also carry out a series of stability tests under fixed temperature of 65 °C and continuous 1‐sun illumination in a nitrogen atmosphere (Figure 4c,d). We first fit the post “burn‐in” section of the PCE to a straight line and extrapolate the curve back to zero time to obtain the *t* = 0 efficiency.^[^ ^61^, ^62^ ^]^ Under constant thermal stability test, the P3HT‐COOH (SA) and P3HT‐COOH (SP) devices showed decent thermal stability with *T*_80_ of 470 and 250 h. In contrast with PEDOT:PSS (SP) device, it quickly reached *T*_80_ lifetime with less than 83 h and completely nonfunction at 170 h. Under light‐soaking aging tests, we determined the lifetime to 80% degradation (*T*_80_) from this *t* = 0 post burn‐in efficiency for the P3HT‐COOH (SA) devices to be 220 h, which is nearly 11 times longer than the *T*_80_ lifetime of the PEDOT:PSS (SP) devices (20 h). However, all of these three devices have short *T*_80_ life time under constant light stress which suggest the degradation mechanism is different than humidity and heat stress which detail discussion can be found in the Supporting Information. Here, the P3HT‐COOH (SA) device demonstrated a much robust and stable performance among P3HT‐COOH (SP) and PEDOT:PSS (SP) devices in moisture, thermal and photo stability testing, which is clearly due to the better crystallinity and high quality perovskites layer formation and suppression of the degradation process.^[^ ^63^ ^]^ Further study and improvement are needed for extending the operation lifetime under light. However, the both P3HT‐COOH cells exhibited higher threshold for degradation than spin coated PEDOT:PSS device.

We have demonstrated a new type of polyelectrolyte P3HT‐COOH as a promising HTM for planar PSCs. This conjugated polyelectrolyte has a relatively low‐lying HOMO level with less surface defects and hydrophobicity which facility the perovskite MAPbI_3_ layer growth with high degree of crystallinity. The perovskite solar cells fabricated by P3HT‐COOH as the HTM show substantially improved *V*_OC_ as well as the *J*_SC_ and F.F.. The self‐assembly approach is a simple, materials‐saving, and cost‐effective method to fabricate a uniform and highly reproducible HTM. Such a method enables a homogeneous packing of polymer chains on the ITO surface as a coil‐like structure conformation and forces more carboxylic acid moieties to face toward ITO surface, forming a more organized interface dipole layer. The ordered and defect free P3HT‐COOH layer down‐shifts the HOMO for enhanced *V*_OC_ in device performance and reduces carrier recombination at the interfaces. As a result, both spin coated and self‐assembled P3HT‐COOH cells outperform the conventional PEDOT:PSS cell significantly on the devices’ *V*_OC_ and *J*_SC_, leading to an average PCE of P3HT‐COOH for 19.21 ± 0.66% (self‐assembled) and 17.01 ± 0.47% (spin coated). In addition, due to a large charge recombination resistance, the photovoltage and photocurrent of the P3HT‐COOH (SA) devices remain extremely sensitive to low light intensity (nW–µW range), indicating its potential application as a highly responsive low‐light detection. Finally, we show a greatly extended cell lifetime using the new P3HT‐COOH layer.

## Experimental Section

##### Materials

The P3HT‐COOH with a wight‐average molecular weight, a polydispersity index, and a regioregularity of 59 000 g mol^−1^, 2.4 and 91%, respectively, was purchased from Rieke Metals and used as received. Regioregular P3HT‐COOH was dissolved in *N*,*N*‐dimethylformamide (DMF) solution with different concentrations of 0.005–1.0 mg mL^−1^. The ITO substrate was treated with O_2_ plasma for 10 min and then dipped into the dilute P3HT‐COOH solution for 16 h. Thereafter, the substrate was immersed into DMF bath and sonicated to remove the residues to get a thin coating layer (Figure S16, Supporting Information). The detailed studies on the effect of P3HT‐COOH solution concentration on the performance of P3HT‐COOH (SP) and P3HT‐COOH (SA) are summarized in Tables S3–S4 in the Supporting Information, respectively. Methylammonium iodide (MAI) was synthesized by reacting hydroiodic acid (40 mL, 57% in water, Sigma‐Aldrich) and methylamine (40 mL, 40% in methanol, Junsei Chemical Co. Ltd) in a 250 mL round bottom flask at 0 °C for 4 h (h) with stirring. The precipitate was recovered by evaporating solvents at 50 °C for 1 h with a rotary evaporator. To purify MAI, the products were firstly washed with diethyl ether, then dissolved in methanol, recrystallized from diethyl ether, and finally dried at room temperature in a vacuum oven for 24 h.

##### Device Fabrication

ITO substrates were cleaned with detergent, deionized water, and sonicated with acetone and isopropanol alcohol for 30 min. MAPbI_3_ solution (40 wt%) was prepared by mixing the MAI powders and PbI_2_ (1:1 molar ratio, Alfa‐Aeser) in and adding 2 moles dimethyl sulfoxide (DMSO) as an additive at room temperature for 12 h. For the inverted MAPbI_3_ PSCs with different HTMs, PEDOT:PSS/isopropanol/deionized water solution with a volume ratio of 10:1:1 was spin‐coated on cleaned ITO with 3500 rpm for 40 s and annealed at 140 °C for 10 min. The P3HT‐COOH solution was spin‐coated on ITO substrate at 4500 rpm for 40 s, and dried at 140 °C for 10 min. A 40% MAPbI_3_ solution containing DMSO as additive was spin coated on top of hole transport layer at 4000 rpm for 25 s, and then washed with ether at 6 s, and then dried at 65/100 °C for 4 min and 1 h. Later, the PCBM (25 mg mL^−1^) in chlorobenzene was coated on top of perovskite at 2000 rpm for 30 s to form a 50 nm layer film as electron transporting layer. PEI with 0.1 mg mL^−1^ in methanol was coated at 4000 rpm for 30 s as an interlayer. Finally, a 100‐nm‐thick silver layer was deposited by thermal evaporation to complete the device fabrications.

##### Characterization

The *J–V* characteristics were performed with forward and reverse scan between −0.1 and 1.1 V with a voltage step of 0.01 V and delay time of 10 ms by a Keithley 2400 source meter (Keithley Instrument. Inc.). The photovoltaic performance measurements were conducted under one‐sun intensity (100 mW cm^−2^) using a Xenon‐lamp based solar simulator calibrated with AM1.5G spectrum (Enli Tech.). The intensity of the solar spectrum was calibrated by a SRC2020 monocrystalline silicon solar cell (Enli Tech.). The SEM measurement were performed using a S‐4800 SEM instrument (Hitachi, Japan) at 5 kV. The XRD data of the perovskite were obtained using a Bruker D8 focus powder XRD instrument equipped with Cu‐K*α* radiation (*λ* = 0.154 nm). The UV–vis absorption spectrum was investigated on a Jasco V‐670 spectrometer (Ja Company, Hachioji, Tokyo, Japan). For the environmental storage test, the devices were placed in an environmental chamber TEN‐H40 (temperature: 0–100°C ± 1 °C, Humidity: 25–95% ± 3%) (Tender Scientific Co., Ltd.) at the preset relative humidity and temperature. The active area was defined by a photomask with an aperture of 0.09 cm^2^. The work function was measured by an AC‐2 photoelectron spectrometer (Riken Keiki Instrument. Inc.). The static PL spectra were measured by an Edinburg PLS 920. Time‐resolved PL decay curves were obtained by a time‐correlated single photon counting (TCSPC) setup. The device was illuminated with a 485 nm laser head LDH‐P‐C‐485 (PicoQuant GmbH) controlled by picosecond pulsed diode laser driver PDL 800‐B (PicoQuant GmbH) at frequency 2.5 MHz, with a pulse duration of 400 ns and fluence of ≈25.4 µW cm^−2^. The PL signal was collected using an ultralow noise single photon avalanche detector ID‐100‐50 (Becker & Hickl GmbH). The EIS measurements were performed using a Metrohm Autolab PGSTAT 302N. A Xenon lamp at 100 mW cm^−2^ providing an air mass AM1.5G spectrum and was used as the light source. A small AC perturbation voltage of 15 mV was applied to the devices, and the different output current was measured throughout a frequency range of 1 MHz–1 Hz.

## Conflict of Interest

The authors declare no conflict of interest.

## Supporting information

Supporting Information
