# Supplementary material for: Facile Fabrication of Self‐Assembly Functionalized Polythiophene Hole Transporting Layer for High Performance Perovskite Solar Cells
Source: Adv Sci (Weinh). 2021 Jan 6;8(5):2002718. doi: 10.1002/advs.202002718 (PMC7927620; doi:10.1002/advs.202002718)
Supplement: Supplementary file 1 — Supporting Information [file ADVS-8-2002718-s001.pdf]

© 2020 Wiley-VCH GmbH

ADVANCED SCIENCE
Open Access

Supporting Information

for *Adv. Sci.,* DOI: 10.1002/advs.202002718

Facile fabrication of self-assembly functionalized polythiophene hole transporting layer for high performance perovskite solar cells

Chi-Yuan Chang, Hsin-Hsiang Huang, Hsinhan Tsai, Shu-Ling Lin, Pang-Hsiao Liu, Fang-Chi Hsu, Wanyi Nie, Yang-Fang Chen* and Leeyih Wang**

Supporting Information

Facile fabrication of self-assembly functionalized polythiophene hole transporting layer for high performance perovskite solar cells

Chi-Yuan Chang, Hsin-Hsiang Huang, Hsinhan Tsai, Shu-Ling Lin, Pang-Hsiao Liu, Fang-Chi Hsu, Wanyi Nie, Yang-Fang Chen* and Leeyih Wang**

C. Y. Chang, H. H. Huang, S. L. Lin, P. H. Liu, Prof. Y. F. Chen, Prof. L. Wang
Center for Condensed Matter Sciences, National Taiwan University.
No. 1, Sec. 4, Roosevelt Rd., Taipei 10617, Taiwan.

Prof. L. Wang
Institute of Polymer Science and Engineering, National Taiwan University.
No. 1, Sec. 4, Roosevelt Rd., Taipei 10617, Taiwan.
Email: leewang@ntu.edu.tw

C. Y. Chang, Prof. Y. F. Chen
Department of Physics, National Taiwan University.
No. 1, Sec. 4, Roosevelt Rd., Taipei 10617, Taiwan.
Email: yfchen@phys.ntu.edu.tw

H. H. Huang, P. H. Liu
Department of Material Science and Engineering, National Taiwan University.
No. 1, Sec. 4, Roosevelt Rd., Taipei 10617, Taiwan.

Dr. W. Nie, Dr. H. Tsai
Materials Physics and Application, Los Alamos National Laboratory
Los Alamos, New Mexico 87545, United States.
Email: wanyi@lanl.gov

Prof. F. C. Hsu
Department of Material Science and Engineering, National United University.
1, Lienda, Miaoli 36003, Taiwan.

Keywords: self-assembled, out-of-plane orientation, hole extraction layer

1. The comparison of PCE for different HTM.

In this study, we mainly focus on development of self-assembly technique to provide cost-effective approach for large scale planar cell fabrication, as well as the interconnecting layer needed for tandem solar cell. We agree that the power conversion efficiencies (PCE) of the P3HT-COOH based PSCs are inferior to the state-of-the-art devices based on the PTAA HTM, but PTAA cannot deposit on top of the ITO surface by self-assembled methods as shown in Figure S8 in the Supporting Information. We believe that the self-assembly approach is a simple, materials-saving, and cost-effective method to fabricate a large-area, uniform and highly reproducible HTM for photovoltaics and a promising interconnecting layer on the textured silicon cells to realize the high performance tandem solar cell. Nevertheless, we agreed that PEDOT:PSS might not a good reference HTM for *p-i-n* type PSCs due to the inferior performance, however, the sulfonate (-SO_3H) group on PSS can anchor on ITO substrate through self-assembled technology. That's the mainly reason we choose it as a reference HTM to compare with our P3HT-COOH HTM. The result clearly showed the solar device based on self-assembled PEDOT:PSS exhibited an extremely poor performance and would not be further discussed in the manuscript. The *J-V* curve of the PSCs using PEDOT:PSS (SA) method is shown in the Figure S9 in the Supporting Information.

In addition, we fabricated PSCs with PTAA as HTM. However, we want to note that according to a previous literature published by Deng et al,[1] the perovskite solution spread on the ITO/PTAA substrate, but shrunk quickly during drying, leaving most of the area uncovered. To improve the wettability of perovskite precursor solution on the PTAA layer, the PTAA-coated ITO substrates were pre-treated by spin-coating 80 μl of DMF at a speed of 4,000 rpm for 15 s prior to perovskite films deposition.[2,3] Briefly, the performance of our PTAA devices are summarized in Figure S10 in the Supporting Information. The wetting issues still remaining, even the PTAA film was pre-wetted by DMF. Compare to our studied devices, we introduce a

facile method to self-assemble a new functionalized polythiophene (P3HT-COOH) monolayer on the conducting oxide electrodes and serve as hole-transporting layer in PSCs with high efficiency, reproducible and stable device performance. We believe that our demonstration broadly impacts the perovskite photovoltaic scientific and industrial research community. We also envision our new self-assembled HTM to be a promising interconnecting layer for the tandem solar cell, where conformal coating on the textured surface is essential for high performance tandem cell.

2. The impact of bottom HTM layer on the device stability.

Degradation under humid condition can be affected by various factors: the perovskite material degradation, moisture absorbed by interface layer that expedite the degradation.[4,5] Therefore, both bottom layer and top layer can affect the perovskite device lifetime under humid condition. In addition, as shown from the all other stability test results and the high performance of the PSCs, the effect of the perovskite crystallinity and crystal orientation is much more pronounced than that of perovskite grain size. This is implying that the larger grain size is one of the contributing factors, however, it does not explain all of the improvements found in P3HT-COOH (SA) films. Moreover, the P3HT-COOH (SA) device exhibits much better crystallinity and high-quality perovskites layer formation in this study which suppressed the degradation process and demonstrated a much robust and stable performance among P3HT-COOH (SP) and PEDOT:PSS (SP) devices under the same testing conditions of moisture, heat, and photostability.

3. Ion migration.

It has been reported that the diffusion of metal ions in ITO contact (indium and tin) into the perovskite layer can significantly degrade the PCE.[6] The ion migration process is normally assisted by vacancy or defect. When using PEDOT:PSS as HTM, the hydrophilic and acidic -SO_3H groups on PSS may induce a faster water absorption and the ITO electrode corrosion

which accelerated the ion migration,[5] thus resulting in rapid degradation of PCE. In contrast, the carboxylic acid anchoring groups not only can self-assembly on ITO surface as HTL but also can passivate the ITO surface and suppressing the ion migration.

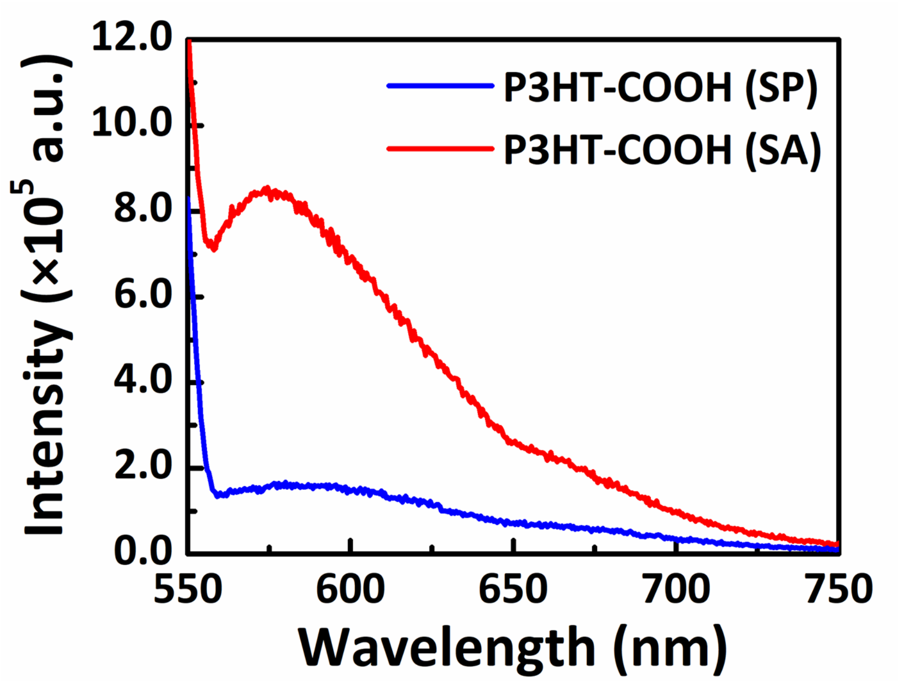

Figure S1. Steady state photoluminescence spectra of spin-coated (SP) and self-assembled (SA) P3HT-COOH layers on ITO substrate.

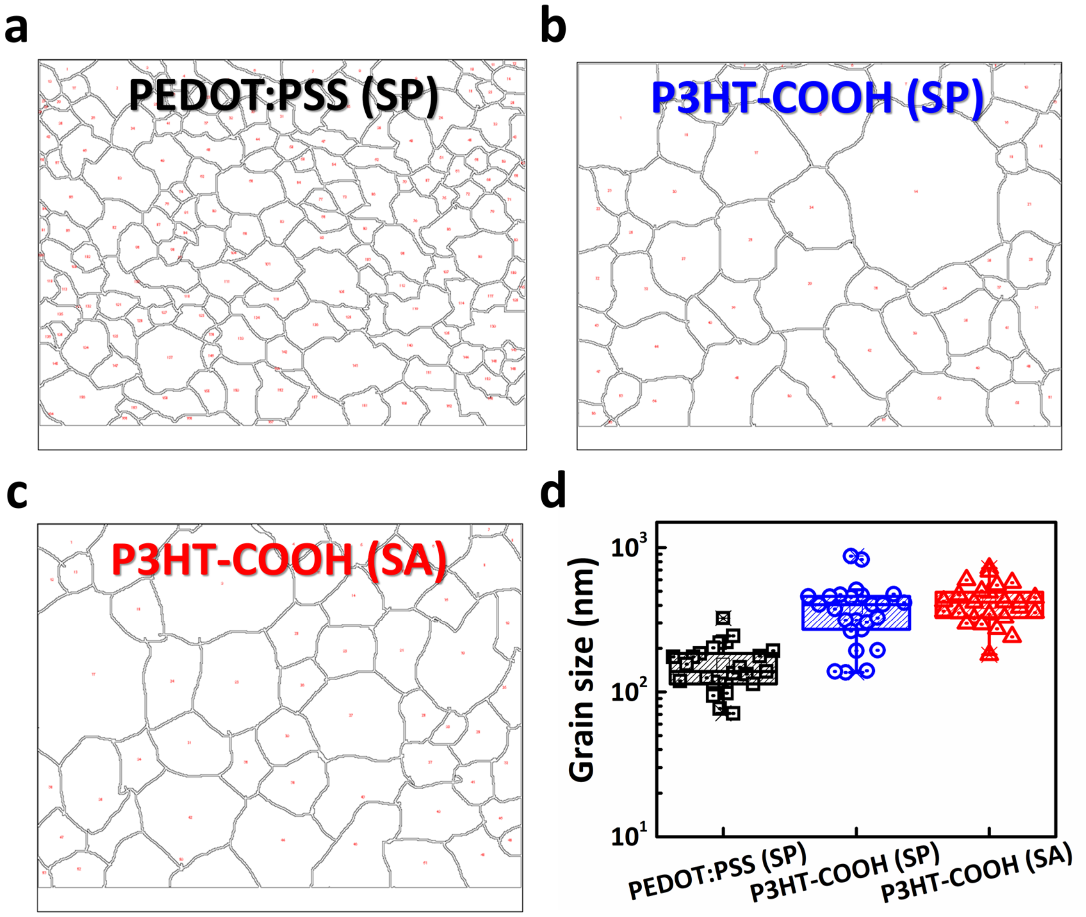

Figure S2. The graph of $MAPbI_3$ films grown on (a) PEDOT:PSS (SP) (b) P3HT-COOH (SP) and (c) P3HT-COOH (SA) layers by ImageJ for determination of the (d) perovskite's average grain size.

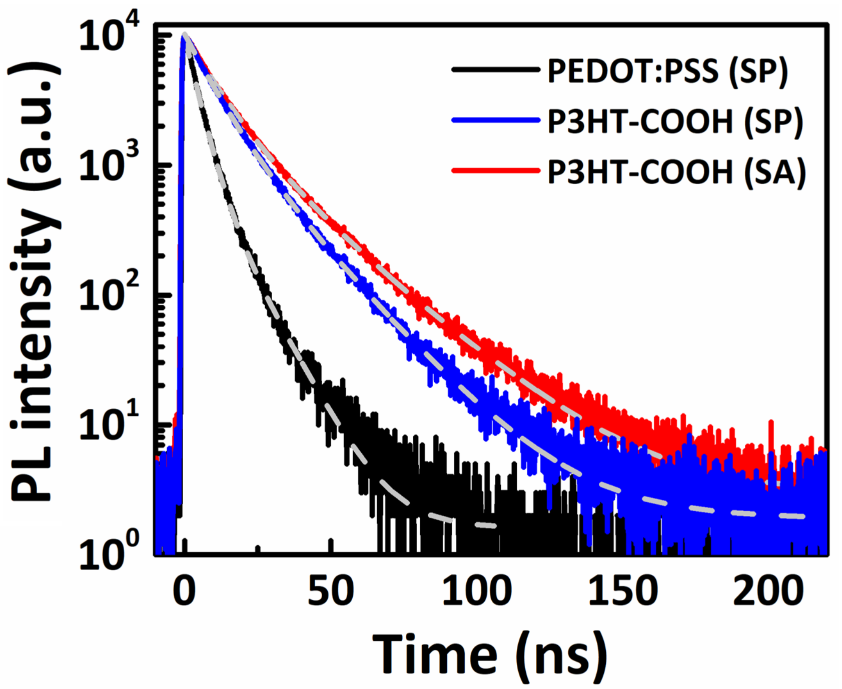

Figure S3. Time-resolved photoluminescence decay curves of $MAPbI_3$ films grown on PEDOT:PSS, spin-coated P3HT-COOH and self-assembled P3HT- COOH films. The lifetime of excitons created in the perovskites on three different substrates decreases in the order: P3HT-COOH (SA) > P3HT-COOH (SP) > PEDOT:PSS, which is consistent with observations in the SEM images.

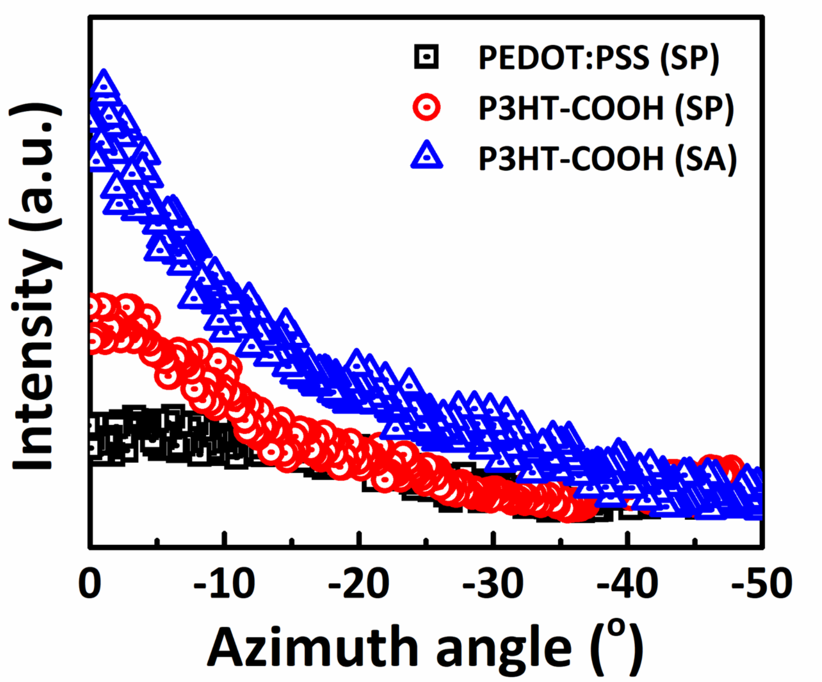

Figure S4. Azimuthal profiles for the $q \approx 1.00$ Å^{-1} reflection peak for perovskite prepared on different HTMs. (i.e., intensity vs azimuth χ, where χ is the azimuth of q_z and q_{xy} in scattering vector polar coordinates with positive q_z at $\chi = 0°$ and positive q_{xy} at $\chi = 90°$). These results suggest that the crystal orientation of the perovskite grown on P3HT-COOH (SA) surface is partially oriented along out-of-plane direction, indicating the crystal domains are parallel to the substrate surface.

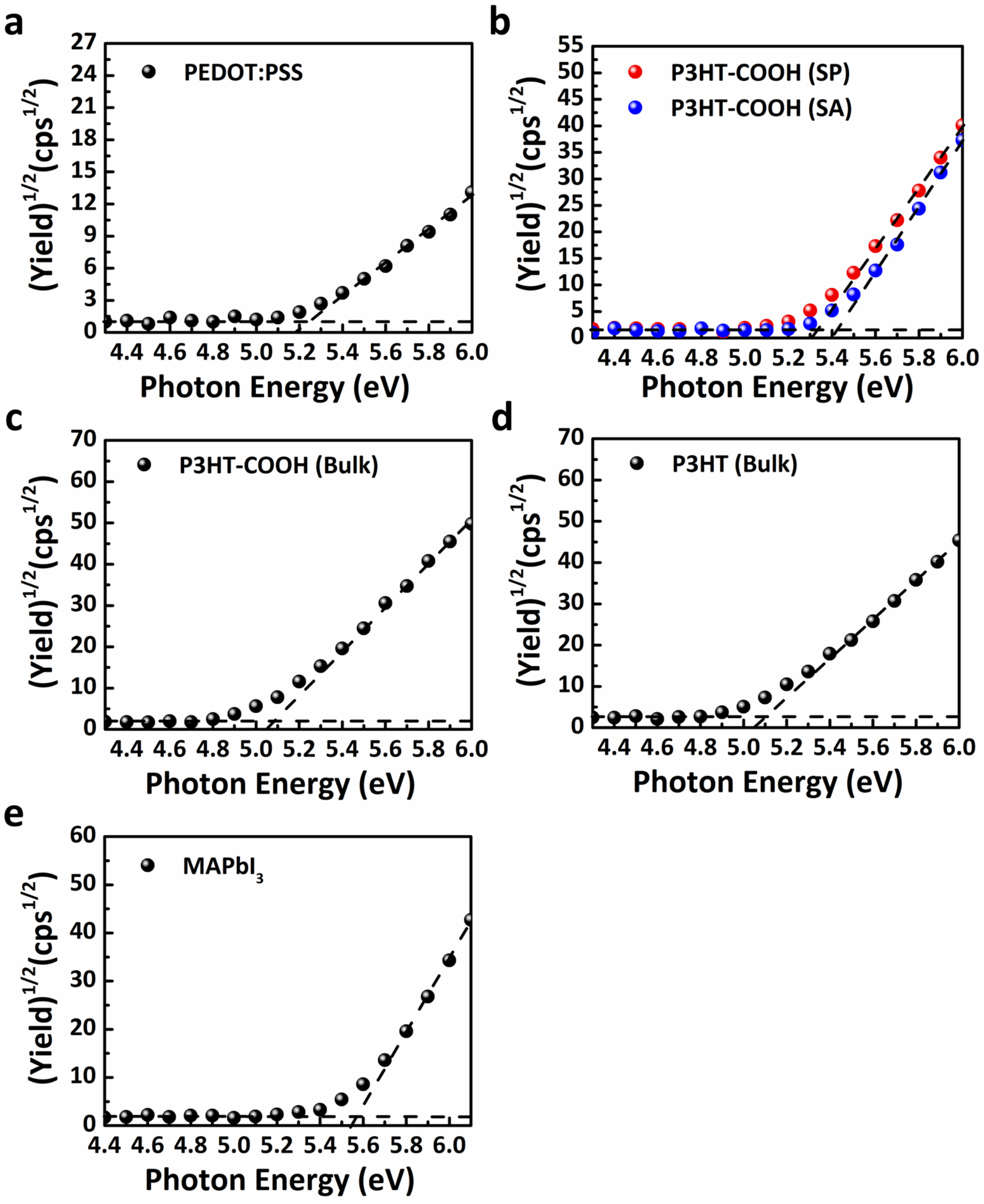

Figure S5. Photoelectron spectra results for (a) PEDOT:PSS (b) spin-coated (SP) and self-assembled (SA) P3HT-COOH, (c) bulk P3HT-COOH, (d) bulk P3HT and (e) $MAPbI_3$ on ITO substrate. The resulting photoelectron spectra and the I_p values determined from the photoemission threshold energy are listed in Table S1 in the Supporting Information.

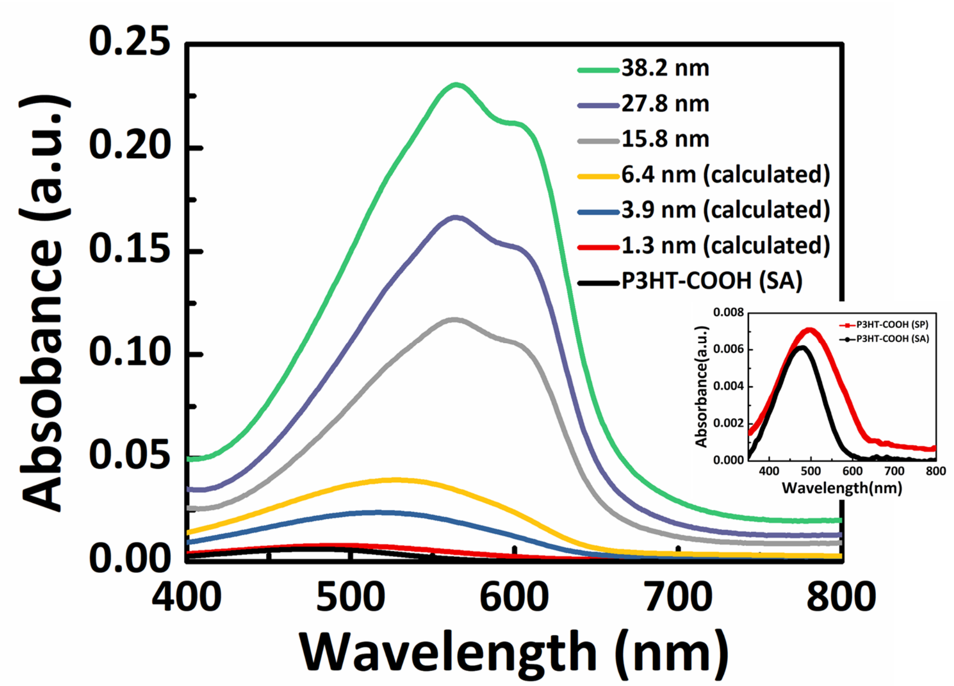

Figure S6. UV-vis absorbance spectra of P3HT-COOH with various film thickness. The relatively thick film exhibits an absorption peak at ~565 nm with a vibronic absorption shoulder at ~603 nm. As the film thickness decreases, the shoulder band becomes ambiguous and the absorption signatures are broadened and blue-shifted towards higher energies, implying the formation of a less aggregated structure and a more coil-like chain conformation that down-shifts the HOMO energy level. The optimal thicknesses of SP and SA P3HT-COOH layers, estimated through the Beer-Lambert-law approach, are around 1.3 and 1.0 nm, respectively. The value of 1.0 nm is close to the length of hexyl carboxylic acid side chain, suggesting the P3HT-COOH (SA) forms a monolayer on top of ITO using the carboxylate as anchoring group. This finding demonstrates that SA route is an effective and simple approach to lower the I_p of conjugated homopolymers without incorporating electron-deficient units into polymer backbone through complicated synthetic steps.

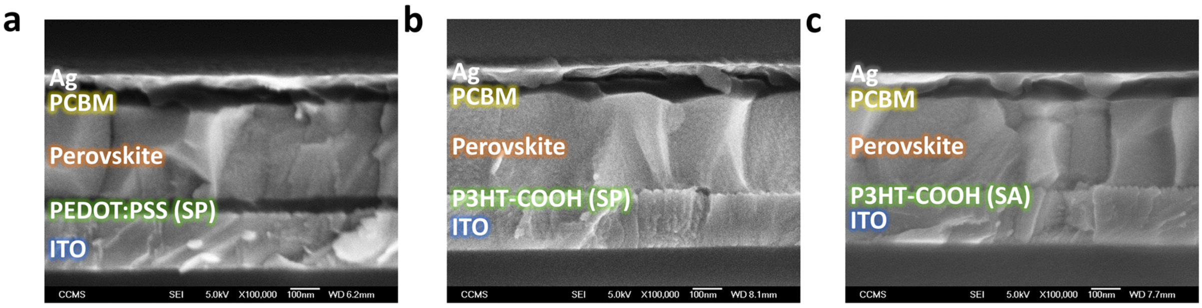

Figure S7. Cross-sectional SEM images of $MAPbI_3$ films deposited on (a) PEDOT:PSS (SP), (b) spin-coated (SP) P3HT-COOH, (c) self-assembled (SA) P3HT-COOH substrates.

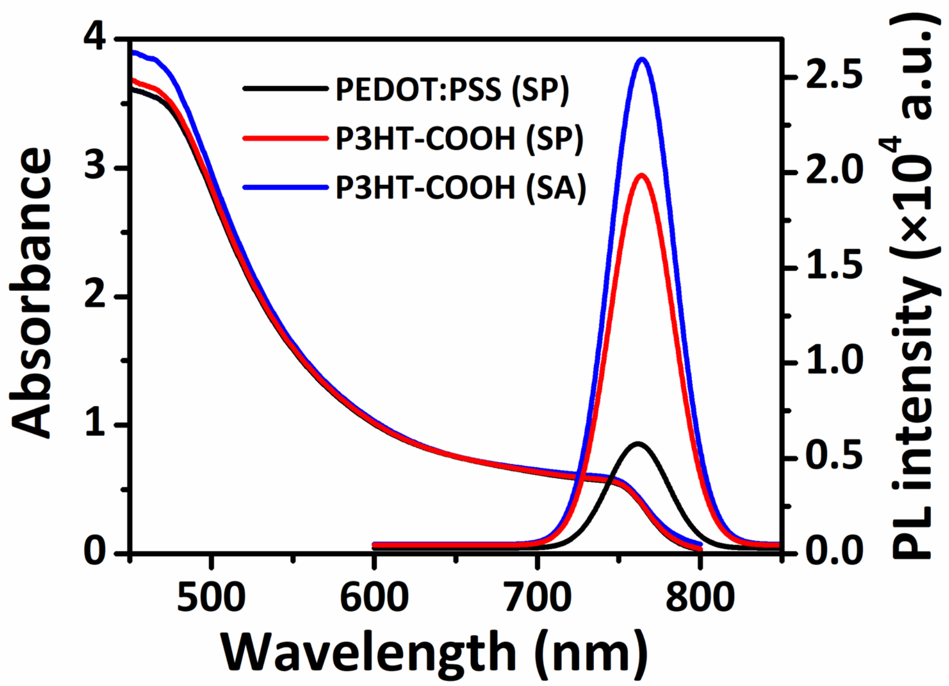

Figure S8. The ultraviolet-visible absorption and steady-state PL spectra of pervoskite grown on different HTMs.

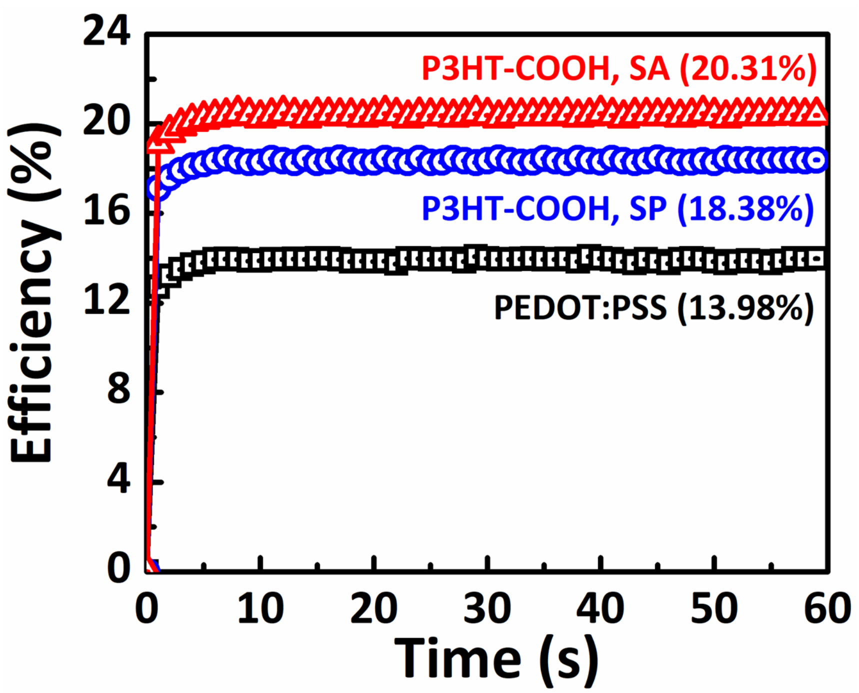

Figure S9. The steady-state output of the PSCs using multiple approach for fabrication of HTM, respectively.

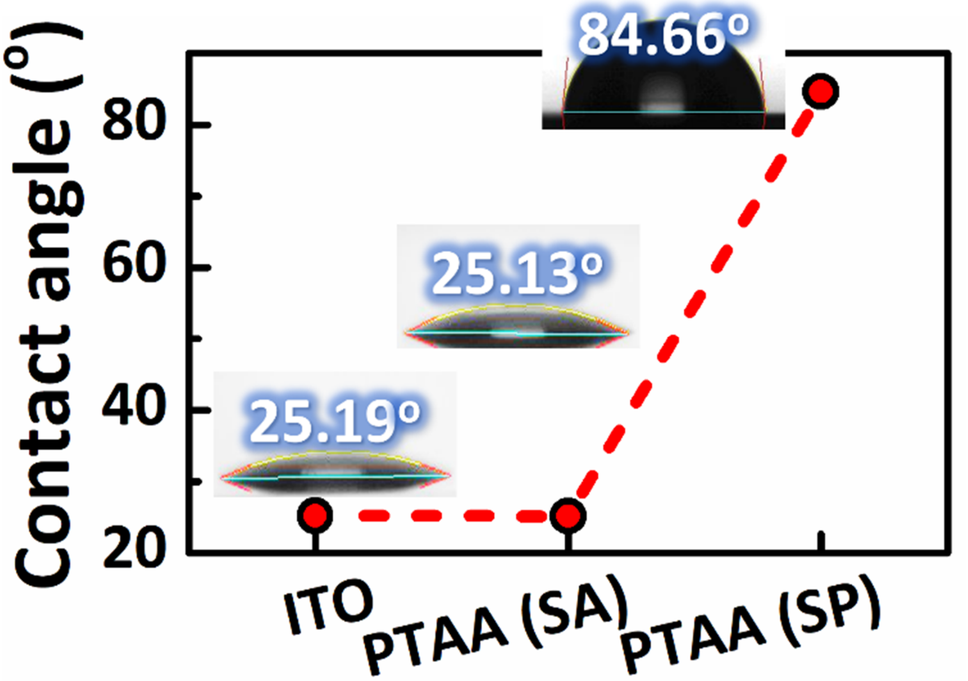

Figure S10. Contact angle photographs of DI water on various PTAA fabricated by different methods (self-assembly (SA) and Spin coating (SP)).

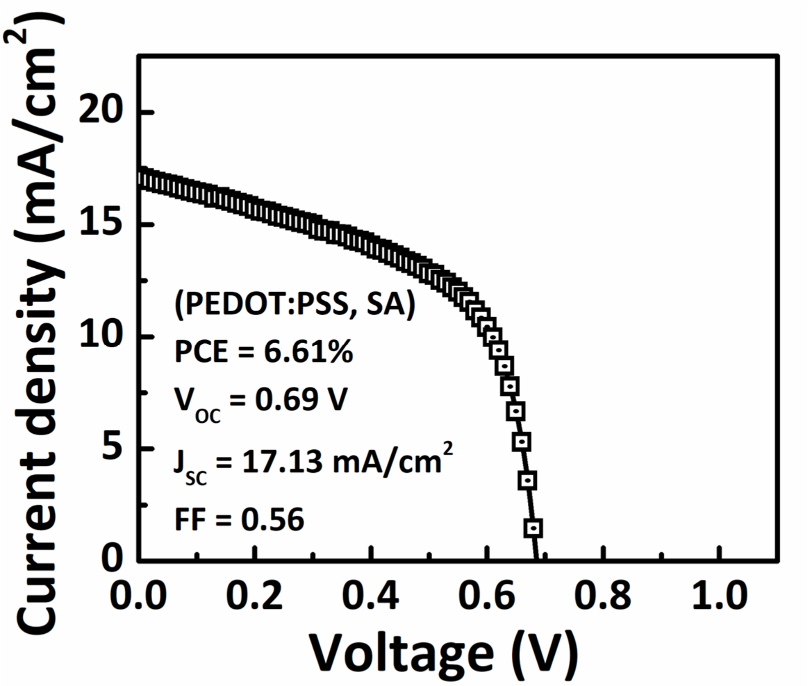

Figure S11. The *J-V* curves of the PSCs using PEDOT:PSS (SA) method.

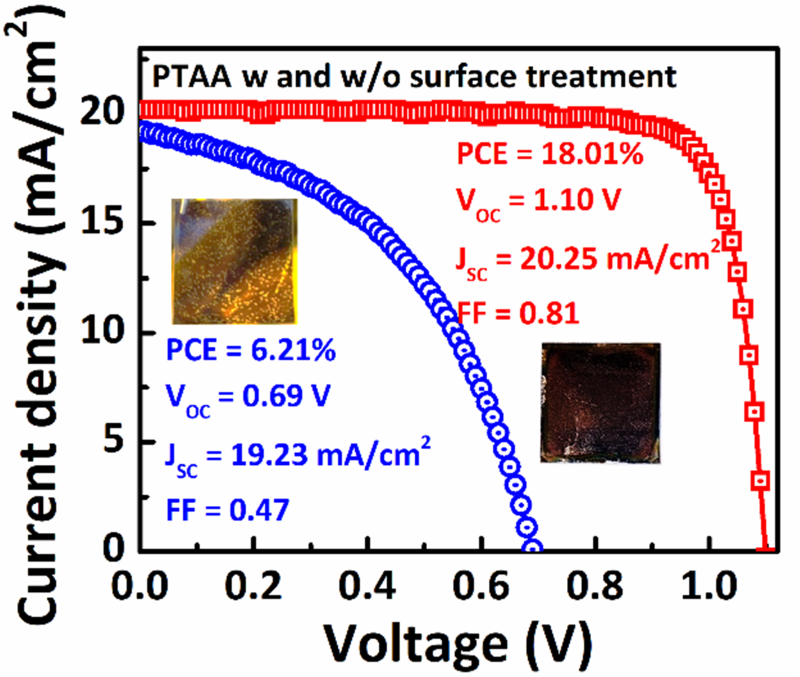

Figure S12. *J-V* curves of PSCs using PTAA as HTM prepared by SP method with and without surface treatment.

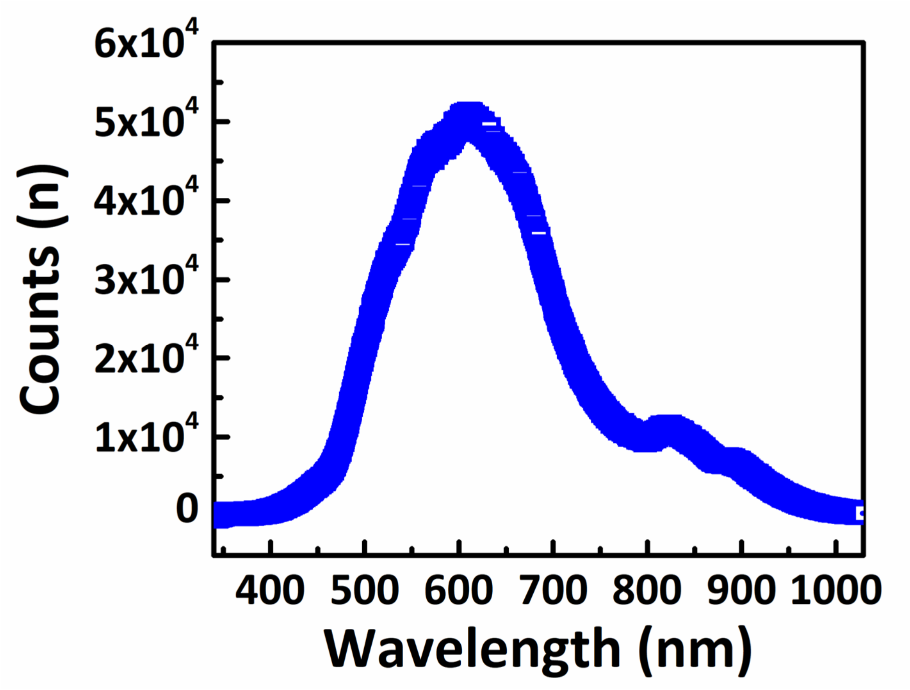

Figure S13. Spectrum characteristic of LED lamp.

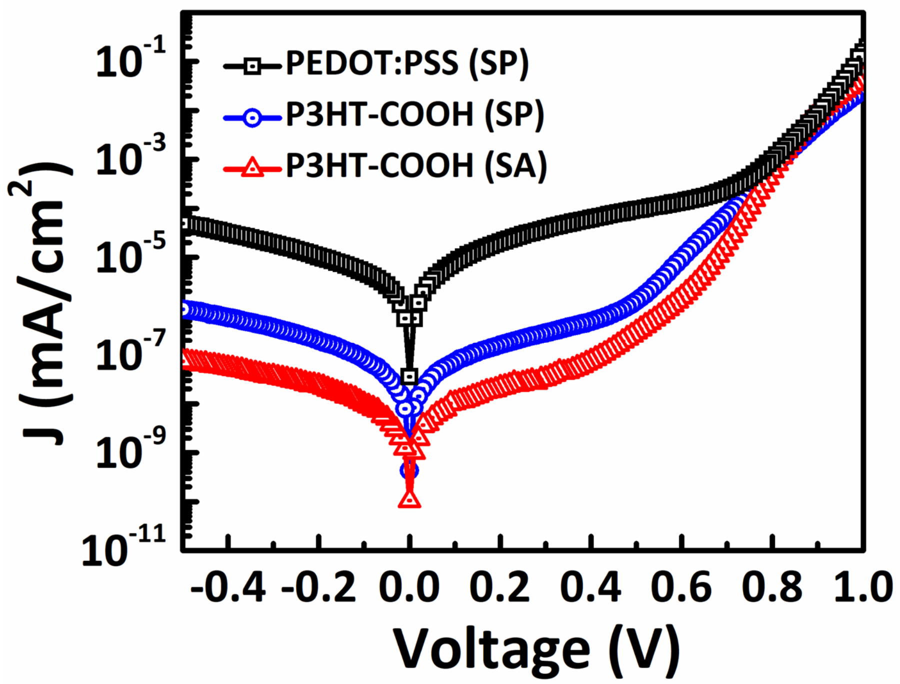

Figure S14. Dark *J-V* curves of $MAPbI_3$ films grown on PEDOT:PSS (SP), P3HT-COOH (SP) and P3HT- COOH (SA) films.

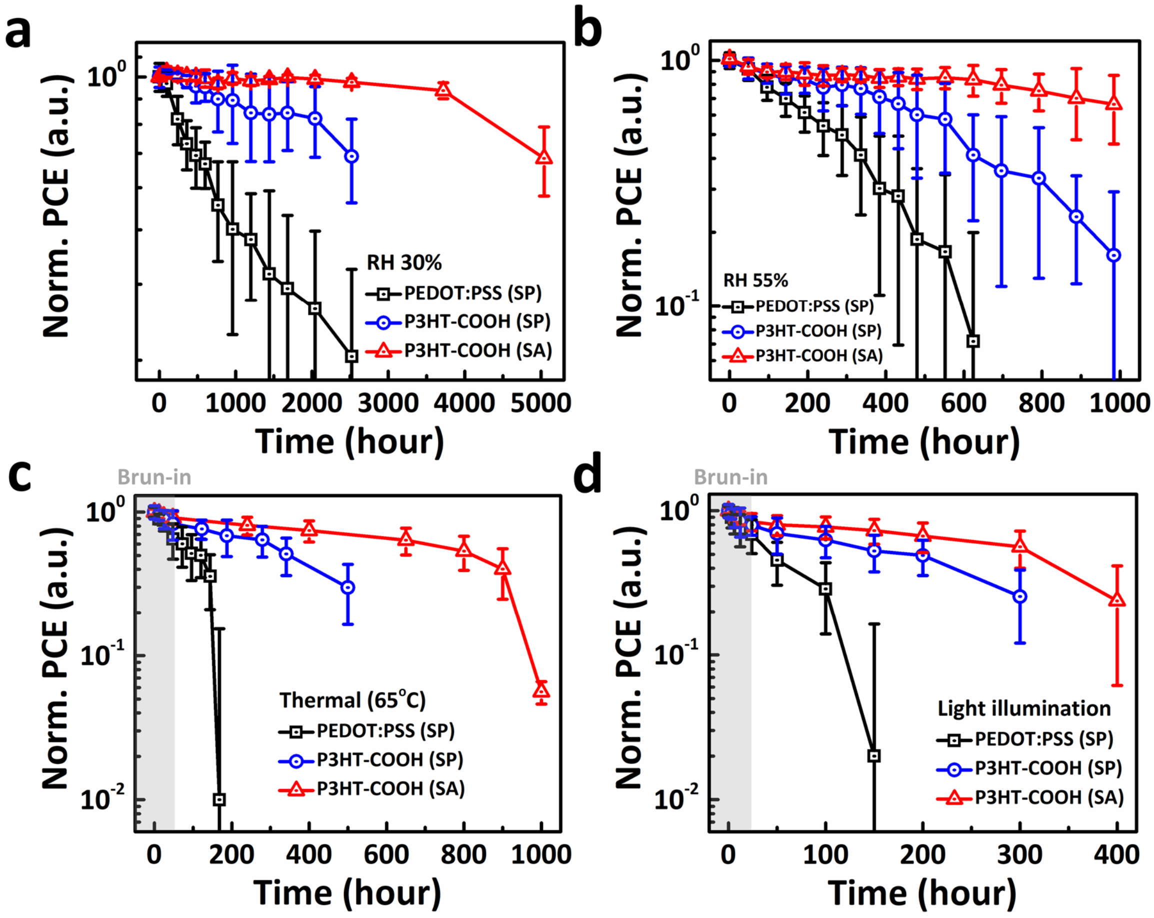

Figure S15. Stability test of PSCs with various HTMs stored under (a) 30% and (b) 55% relative humidity. (c) Normalized PCE of devices under thermal testing at 65°C in glove box and (d) photostability test under constant 1-sun illumination with a full spectrum AM1.5G source. All data were collected over ten devices for statistics.

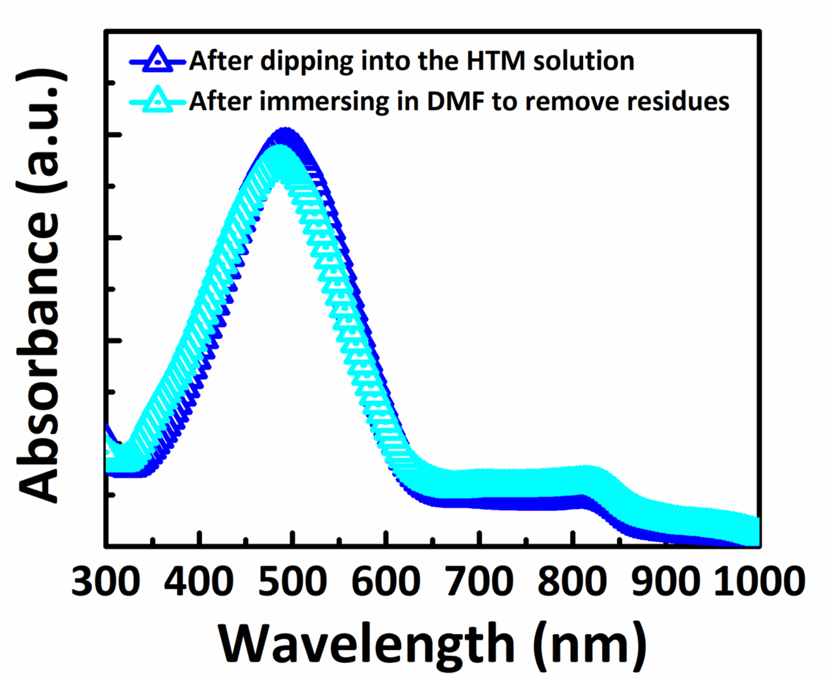

Figure S16. The UV-vis spectra of P3HT-COOH on ITO substrate and after immersing in DMF solution to remove residues.

Table S1. Ionization energy (I_p or W_f for ITO), light absorption bandgap (E_g^{opt}), and electron affinity (EA) values of various materials on ITO substrates.

Materials	I_p [eV]	E_g^{opt} [eV]	EA [eV]
P3HT (Bulk)	5.11	2.00	3.11
P3HT-COOH (Bulk)	5.10	2.00	3.10
P3HT-COOH (SP)	5.32	1.97	3.35
P3HT-COOH (SA)	5.42	2.16	3.26
PEDOT:PSS	5.25	-	-
$MAPbI_3$	5.58	1.58	4.00
ITO	5.18	-	-

Table S2. Performance parameters of devices with respect to different HTMs.

Device	V_{OC} (V)	J_{SC} (mA/cm^2)	*FF* (%)	PCE (%)	R_S (Ω cm^2)	R_{sh} (kΩ cm^2)
PEDOT:PSS	0.86 ± 0.06	18.11 ± 0.93	0.77 ± 0.05	11.71 ± 1.23	2.53 ± 0.35	2.28 ± 1.56
(SP)	1.04 ± 0.01	20.68 ± 0.34	0.78 ± 0.01	17.01 ± 0.47	2.71 ± 0.49	2.43 ± 1.91
(SA)	1.09 ± 0.01	22.07 ± 0.28	0.81 ± 0.01	19.21 ± 0.66	2.60 ± 0.25	3.36 ± 3.28

Table S3. Photovoltaic parameters of PSCs with a spin-coated (SP) hole extraction layer prepared from various concentrations of P3HT-COOH.

P3HT-COOH (mg/mL)	V_{OC} (V)	J_{SC} (mA/cm^2)	*FF* (%)	PCE (%)
0.5	1.05 ± 0.01	20.37 ± 0.24	0.77 ± 0.01	16.48 ± 0.39
1.0	1.04 ± 0.00	20.68 ± 0.34	0.78 ± 0.01	17.01 ± 0.47
3.0	1.01 ± 0.01	19.32 ± 0.53	0.79 ± 0.01	15.39 ± 0.52
5.0	1.01 ± 0.03	19.23 ± 0.28	0.79 ± 0.02	15.21 ± 0.21

Table S4. Photovoltaic parameters of perovskite solar cells with a self-assembled (SA) hole extraction layer prepared from various concentrations of P3HT-COOH.

P3HT-COOH (mg/mL)	V_{OC} (V)	J_{SC} (mA/cm^2)	*FF* (%)	PCE (%)
0.005	1.05 ± 0.01	20.53 ± 0.47	0.80 ± 0.01	17.29 ± 0.43
0.05	1.07 ± 0.01	21.14 ± 0.21	0.79 ± 0.01	17.87 ± 0.23
0.1	1.09 ± 0.01	22.07 ± 0.28	0.81 ± 0.01	19.21 ± 0.66
0.5	1.07 ± 0.01	21.26 ± 0.16	0.79 ± 0.01	17.86 ± 0.16
1.0	1.07 ± 0.01	21.16 ± 0.16	0.78 ± 0.01	17.70 ± 0.21

Reference

[1] Y. H. Deng, X. P. Zheng, Y. Bai, Q. Wang, J. J. Zhao, J. S. Huang, *Nat. Energy* **2018**, *3*, 560.

[2] S. Yang, S. Chen, E. Mosconi, Y. Fang, X. Xiao, C. Wang, Y. Zhou, Z. Yu, J. Zhao, Y. Gao, F. De Angelis, J. Huang, *Science* **2019**, *365*, 473.

[3] D. Luo, W. Yang, Z. Wang, A. Sadhanala, Q. Hu, R. Su, R. Shivanna, G. F. Trindade, J. F. Watts, Z. Xu, T. Liu, K. Chen, F. Ye, P. Wu, L. Zhao, J. Wu, Y. Tu, Y. Zhang, X. Yang, W. Zhang, R. H. Friend, Q. Gong, H. J. Snaith, R. Zhu, *Science* **2018**, *360*, 1442.

[4] Z. Li, K. Cao, J. Li, X. Du, Y. Tang, B. Yu, *Org. Electron.* **2020**, *81*, 105675.

[5] P. Cheng, X. Zhan, *Chem. Soc. Rev.* **2016**, *45*, 2544.

[6] C. T. Howells, S. Saylan, H. Kim, K. Marbou, T. Aoyama, A. Nakao, M. Uchiyama, I. D. W. Samuel, D.-W. Kim, M. S. Dahlem, P. André, *J. Mater. Chem. A* **2018**, *6*, 16012.
